# PASSING ON THE SILVER SPOON: THE ROLE OF EARLY LIFE CIRCUMSTANCES ON DOWNWARD INTERGENERATIONAL FINANCIAL TRANSFERS

**DOI:** 10.1093/geroni/igac059.2333

**Published:** 2022-12-20

**Authors:** Kent Jason Cheng

**Affiliations:** Syracuse University, Syracuse, New York, United States

## Abstract

Childhood circumstances are known to etch an indelible influence on individual outcomes across the life course such as education, income, and health. Yet relatively little is known about how early life exposures influence family outcomes. This study aims to determine how childhood exposures are associated with inter vivos downward intergenerational financial transfers among older adults aged 51-85 with at least one surviving adult child. Using cumulative (dis)advantage theory, I hypothesized that experiences in childhood shape intergenerational transfers patterns – with early-life advantage being able to provide more transfers than their disadvantaged counterparts. I used data from the US Health and Retirement Study waves 1998 to 2018 (n=32,095 individuals, 169,316 person-years) and estimated random effects models. The index of childhood socioeconomic status was constructed by adding the following dichotomized indicators: poor to fair family socioeconomic status, mother / father having less than HS education, father working for service sector, and moved due to financial difficulty. Downward transfers were defined as whether the respondent gave money, helped pay bills, or covered certain costs to children or grandchildren worth ≥US$ 500. The unadjusted model revealed that the probability of providing downward transfers among those with 1 and 2+ childhood socioeconomic status disadvantage was 5.4% and 3.8% less than those without disadvantage. The inverse association of early life disadvantage and downward transfers were preserved when basic demographic controls and household income and wealth were controlled for, although the magnitude slightly declined (4.0% and 3.1% lower probability for those with 1 and 2+ misfortunes, respectively).

